# Impacts of rising sea temperature on krill increase risks for predators in the Scotia Sea

**DOI:** 10.1371/journal.pone.0191011

**Published:** 2018-01-31

**Authors:** Emily S. Klein, Simeon L. Hill, Jefferson T. Hinke, Tony Phillips, George M. Watters

**Affiliations:** 1 Antarctic Ecosystem Research Division, Southwest Fisheries Science Center, National Marine Fisheries Service, National Oceanic and Atmospheric Administration, La Jolla, California, United States of America; 2 Farallon Institute, Petaluma, California, United States of America; 3 British Antarctic Survey, Natural Environment Research Council, Cambridge, United Kingdom; Department of Agriculture and Water Resources, AUSTRALIA

## Abstract

Climate change is a threat to marine ecosystems and the services they provide, and reducing fishing pressure is one option for mitigating the overall consequences for marine biota. We used a minimally realistic ecosystem model to examine how projected effects of ocean warming on the growth of Antarctic krill, *Euphausia superba*, might affect populations of krill and dependent predators (whales, penguins, seals, and fish) in the Scotia Sea. We also investigated the potential to mitigate depletion risk for predators by curtailing krill fishing at different points in the 21^st^ century. The projected effects of ocean warming on krill biomass were strongest in the northern Scotia Sea, with a ≥40% decline in the mass of individual krill. Projections also suggest a 25% chance that krill biomass will fall below an established depletion threshold (75% of its unimpacted level), with consequent risks for some predator populations, especially penguins. Average penguin abundance declined by up to 30% of its unimpacted level, with up to a 50% chance of falling below the depletion threshold. Simulated krill fishing at currently permitted harvest rates further increased risks for depletion, and stopping fishing offset the increased risks associated with ocean warming in our model to some extent. These results varied by location and species group. Risk reductions at smaller spatial scales also differed from those at the regional level, which suggests that some predator populations may be more vulnerable than others to future changes in krill biomass. However, impacts on predators did not always map directly to those for krill. Our findings indicate the importance of identifying vulnerable marine populations and targeting protection measures at appropriate spatial scales, and the potential for spatially-structured management to avoid aggravating risks associated with rising ocean temperatures. This may help balance tradeoffs among marine ecosystem services in an uncertain future.

## Introduction

Marine ecosystems provide important provisioning, regulatory and cultural services, but climate change may threaten their sustainable delivery (e.g., [1–3]). The impacts of climate change can propagate through food webs as changes affecting one species also influence its competitors, predators, and prey [4]. This can further alter the various services an ecosystem provides for people, potentially motivating the re-assessment of tradeoffs between these services [1]. In addition, the consequences of climate change may be compounded by fishing or other human uses. Reducing such uses (and possibly accepting a cost in other ecosystem services, such as provisioning) might help compensate for the negative impacts of climate change on marine biota (e.g. reduced population sizes). For example, where climate change increases the risks of negative outcomes associated with exploited and overexploited fisheries, these risks may be offset by reducing overall catch [5].

**Fig 1:**
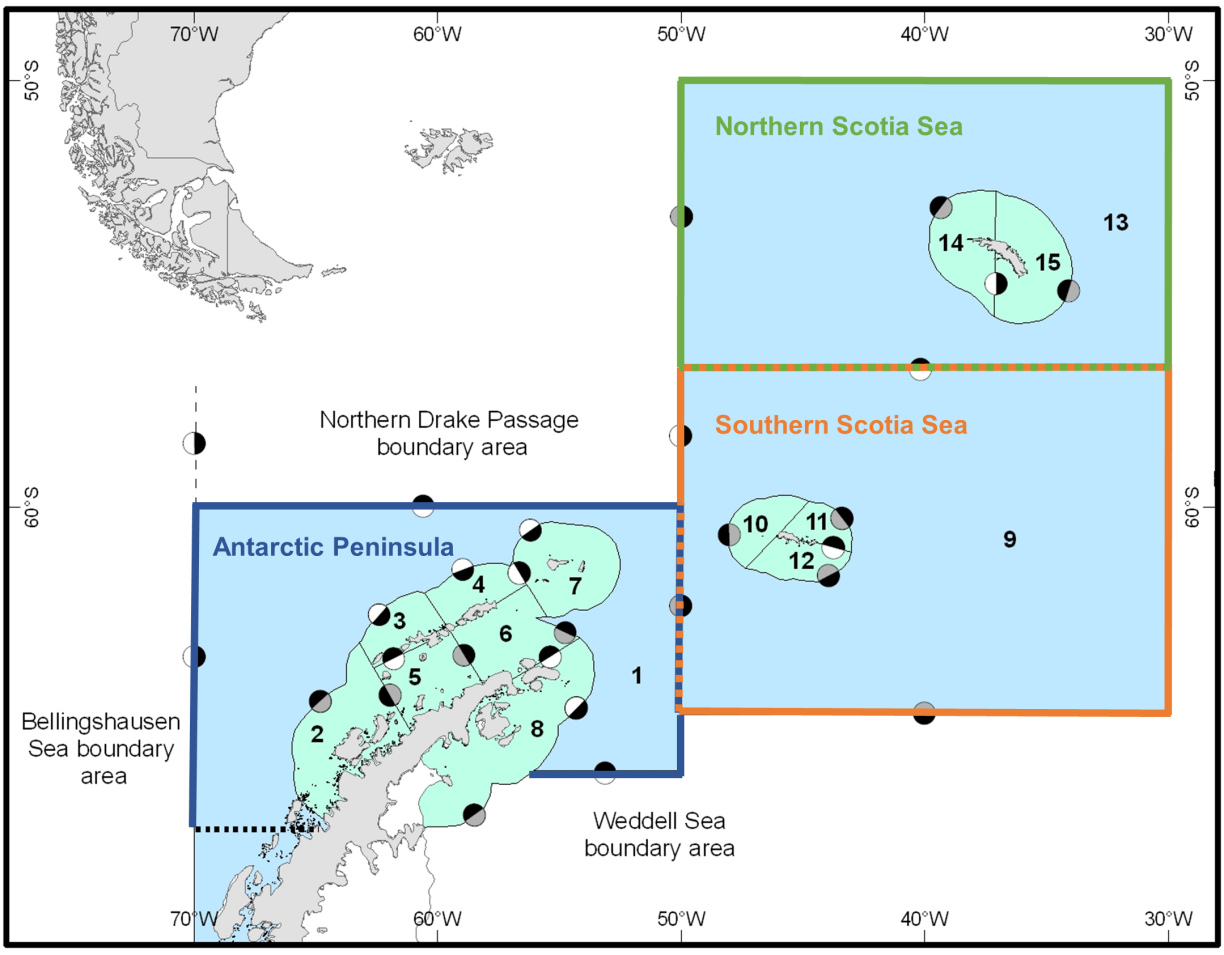
Spatial structure of the Scotia Sea ecosystem model. Small-scale management units (SSMUs) are numbered, and subareas and boundary areas identified (figure adapted from [13]). Part of SSMU 1, south of the dashed black line, was not modelled. Circles denote summer movement of krill between neighboring areas in movement scenarios, and grayscales demonstrate the strength of flow between the areas: black indicating areas receiving dominant inflow, grey for minor inflow, and white circles showing no inflow.

The Scotia Sea, in the southwest Atlantic sector of the Southern Ocean (Fig 1), presents a case study to explore how the potential effects of climate change on an important forage species might affect higher trophic levels, and how fisheries-management actions might interact with broader climate impacts to affect tradeoffs among ecosystem services. The Scotia Sea provides provisioning services via fisheries, including one targeting Antarctic krill, *Euphausia superba*, and cultural services via an array of charismatic predators that are important for ecotourism and of wider conservation concern [6]. These predators include numerous penguins, seals and whales, which feed primarily on Antarctic krill [7]. Several studies suggest that climate change could have negative impacts on the krill population (e.g. [8–10]), although at least two identify potential positive effects [11, 12]. Any negative outcomes are likely to reduce the biomass of krill, with potential consequences for the ecosystem’s associated provisioning and cultural services.

Fisheries managers need to understand how broader climate-change impacts will interact with those of fishing, and this is particularly pertinent when management is tasked with balancing the provision of various, potentially competing, ecosystem services. The Commission for the Conservation of Antarctic Marine Living Resources (CCAMLR) manages fisheries in the Southern Ocean, and its management objectives include ecosystem considerations (e.g., maintaining relationships between species), as well as sustainable use [14–16]. Therefore, regional catch limits, including the highly conservative limit for the Scotia Sea (c. 1% of biomass estimated in 2000), set aside some krill production for krill-dependent predators [15, 16]. However, concerns remain that regional catch limits alone do not mitigate localized risks to those predators [15, 16]. CCAMLR has thus endorsed future management on the basis of “small-scale management units” (SSMUs, Fig 1) [17] and, in the meantime, subdivided the Scotia Sea catch limit amongst four larger subareas, including three that are actively fished and addressed in this study (the Antarctic Peninsula and the northern and southern Scotia Sea, Fig 1)[14, 16]. For CCAMLR, insight into how climate change may affect krill and predator populations across varying spatial scales is of direct relevance for future management. We focus our analysis on the SSMU scale.

Projecting the cumulative, ecosystem impacts of multiple, interacting drivers to understand climate-change outcomes and potential management action is fraught with complexity and uncertainty. Given this, a useful approach is to focus on assessing the marginal impact of a single, well-defined process relative to a reference case (i.e., a case which lacks the impact but is otherwise equivalent) (e.g., [13]). One such process by which climate change might impact the Scotia Sea ecosystem is the effect of increasing sea temperatures on growth rates of Antarctic krill. Hill et al. [10] reported that changes in temperature will likely have spatially variable, but mainly negative impacts on krill growth in the Scotia Sea.

Here we used a minimally realistic (sensu [18]) ecosystem model developed by Watters et al. [13] to explore how the temperature impacts on krill growth estimated by Hill et al. [10] might propagate to dependent predators and the implications of such propagation for management aimed at balancing ecosystem services. We focused on the marginal effects of warming and fishing relative to a reference scenario, which projects what krill biomass and predator abundances might be in the absence of warming and fishing. We further considered whether closures of the krill fishery could help to offset the increased risks posed by warming. To these ends, we asked three connected questions: (i) how might the effects of rising temperature on krill growth affect krill biomass and the abundance of krill predators in the Scotia Sea (Q1); (ii) how do the current, permitted level and spatial distribution of fishing effort affect the ecosystem impacts of rising temperature (Q2); and (iii) can increased ecosystem risks of rising temperature be offset by stopping krill fishing (Q3)?

## Methods

### The model

We used a spatially explicit ecosystem model developed in R [19], described by Watters et al. [13], and with a sensitivity analysis provided by Hill & Matthews [20]. Briefly, this minimally realistic model uses delay-difference equations to describe the dynamics of krill and four krill-dependent predator groups (penguins, seals, whales, and fishes). The model includes two time-steps (seasons) per year, and spatially represents 15 SSMUs in the Scotia Sea (Fig 1). We modeled the post-larval abundance of krill in each SSMU as a function of stochastic recruitment and area-specific mortality and movement. At the beginning of each time step, the numbers of krill in an SSMU are converted to biomass using an estimate of the mean mass per individual krill, *wbar*. In the case where krill have moved into an SSMU from other areas of the model, *wbar* is the abundance-weighted mean mass of the mixture.

Predators and the fishery consume from the area-specific krill biomass estimated at the beginning of each time step, and competition arises when biomass is insufficient to satisfy the combined demand. If the average mass of an individual krill changes because of rising temperatures (see below), this can alter the biomass of krill available.

Currently, the fishery for Antarctic krill operates almost exclusively in the area represented in the ecosystem model, which is divided into the 15 SSMUs (Fig 1; see [17]). Following Watters et al. [13], we treated each predator group breeding in an SSMU as an independent population. The model also includes three boundary areas that supply krill to the SSMUs from the Bellingshausen Sea, Weddell Sea, and northern Drake Passage (Fig 1).

We employed most of the ecological parameters and model structures described by Watters et al. [13] and their approach for addressing key uncertainties in our understanding of the ecosystem. This included the four parameterizations developed by Watters et al. [13], which bracket plausible values for rates of krill movement between SSMUs (no movement and movement as passive drifters) and sensitivities of predators to krill availability (hyperstable and linear). Two elements of the model were revised. First, we updated the distributions and seasonality of foraging effort by penguins, whales, and seals using recent tracking data [21]. Second, we adjusted the model to include time-series variation in the average mass of individual krill (see below).

### Implementing temperature impacts on krill growth

We assessed the outcomes of rising temperatures on krill and krill-dependent predators over the 21^st^ century (2007–2100) based on changes in krill gross growth potential (GGP). We acknowledge that there are other avenues by which temperature change could influence krill predators, but we do not explore those processes here. Krill GGP is defined as the dry mass of an individual krill at the end of the summer growth period divided by its dry mass at the beginning of the season. It is an indicator of the habitat’s ability to support krill growth. Hill et al. [10] used a statistical model to estimate changes in krill GGP in the Atlantic sector of the Southern Ocean based on sea surface temperature (SST), chlorophyll-a concentration, and the initial size of krill. Here, we used their estimates of krill GGP based on an initial length of 40mm (which corresponds with the mean mass of 0.46g assumed by Watters et al. [13]), climatological chlorophyll-a data from SeaWiFS [22], and projected SST output from Coupled Model Intercomparison Project Phase 5 (CMIP5) [23] Representative Control Pathways (RCPs) 8.5 and 2.6 [24]. RCP 8.5 represents a continuous increase in greenhouse gas concentrations resulting in radiative forcing (a key driver of climate change) reaching 8.5 W∙m^-2^ by 2100. RCP 2.6 represents peak radiative forcing of 3.1 W∙m^-2^ around 2045, after which it falls to ~2.6 W∙m^-2^ by 2100. Chlorophyll-a concentrations were kept constant, while SST varied based on the RCPs.

We derived annual, SSMU-scale krill GGP estimates for each RCP from the gridded (1° longitude by 0.5° latitude) estimates of Hill et al. [10]. These SSMU-scale GGP estimates were calculated as weighted averages of the gridded GGP estimates, where the weighting was based on the area of each grid cell contained within each SSMU. For model input, we divided these SSMU-scale, annual GGP estimates by the value for the previous year, setting the initial quotient (for 2007) to 1, to give a SSMU-specific index, *δ*, of the relative change in mean mass of individual krill over each summer growth season. We set this index to 1 for each winter season, assuming no growth or shrinkage during winter. We then adapted the model of Watters et al. [13] to vary the mean mass of krill, *wbar*, for each time step, *t*, in each area, *j*, as:

$$wbar_{t+1,j}\;=\;wbar_{t,j}\;\times \;\delta_{t,j}$$

where *wbar*_*1*,*j*_ = 0.46g. Recall that *wbar* is used to convert krill abundance from numbers to biomass, and that any given *wbar*_*t*,*j*_ is estimated as the mean mass of the final mixture of resident krill and any immigrant krill that arrived in the SSMU during the time step *t*-1. The updated *wbar*_*t*,*j*_ is then subject to its current, SSMU-specific *δ*_*t*,*j*_. That is, the krill that arrive in an SSMU from other locations are converted to biomass via the *δ*_*t*,*j*_ for the current SSMU at the beginning of the following time-step. Thus, the model preserves spatial effects of GGP on krill mass under krill-movement parameterizations.

### Implementing fishing

The model includes several “fishing options” that define candidate management strategies based on both the overall level of catch and the proportional distribution of catches among the SSMUs (see [13]). We used the “historical fishing” option to define a management strategy based on current catch limits for the krill fishery. We specified an overall harvest rate throughout the model arena of 0.01, which represents the current catch limit of 620,000 t per year in the Scotia Sea [16]. To represent the spatial subdivision of this catch limit into the three subareas currently used in management [16], we specified that no more than a set proportion of the overall harvest could be removed from each of SSMUs 1–8 combined (25% limit), SSMUs 9–12 combined (45% limit), and SSMUs 13–15 combined (45% limit). We simulated the spatial distribution of catches among SSMUs based on the average distribution of actual catches between 2009 and 2015, first among SSMUs annually, and then within each SSMU seasonally (S1 Table).

### Simulations, scenarios, and risk metrics

Following Watters et al. [13], we used a Monte Carlo approach to evaluate whether the effects of warming on krill growth might have consequent risks for krill-dependent predators, and if fishing has an additional influence. Throughout the remainder of this paper, we refer to individual runs of the model as simulations; each simulation was run with a random vector of annual krill recruitments over the 93-year period from 2007–2099, as per [13]. We refer to sets of simulations as scenarios, which are defined by a specific combination of GGP estimates and fishing, and include all four of the model parameterizations described previously. Therefore, to bracket uncertainty, we ran 1001 simulations per model parameterization, resulting in a total of 4004 simulations per scenario.

We established a reference scenario (“Reference”, Table 1) that did not include the effects of warming or fishing. We used this reference scenario as the basis for estimating the mean marginal impacts (shortened to “marginal impacts” for brevity) of warming, fishing, and both combined. We computed these marginal impacts (see below) as ratios of average krill biomass or predator abundance over the last 30 years of each simulation (i.e., 2069–2099) relative to those from the reference scenario. If there was no marginal impact, this ratio equaled one. If there was a marginal impact, these ratios were greater or less than one, respectively, depending on whether impacts were positive (increased biomass or abundance relative to the reference scenario, >1) or negative (decreased biomass or abundance, <1). The ratios also provided a standardized index for comparing results across SSMUs, which vary greatly in area and the numbers of animals breeding and feeding within them.

**Table 1:** Summary of model scenarios.

Assessment	Scenario	Climate-change pathway	Fishing?	Purpose
Reference	“Reference”	None	No	*Model outcomes in an unimpacted ecosystem for use in calculating the marginal impacts of simulated warming and fishing*.
Effects of rising temperature on krill growth (Q1)	“RCP26”	RCP 2.6	No	*Model outcomes for the effects of warming on krill growth under RCP 2*.*6*.
	“RCP85”	RCP 8.5	No	*Model outcomes for the effects of warming on krill growth under RCP 8*.*5*.
Effect of fishing on impacts associated with rising temperature (Q2)	“Fish”	None	Yes, full simulation period	*Assessment of the effects of fishing in the absence of warming impacts on krill growth*.
	“RCP26_Fish”	RCP 2.6	Yes, full simulation period	*Assessment of cumulative impacts of warming and fishing*.
	“RCP85_Fish”	RCP 8.5	Yes, full simulation period	*Assessment of cumulative impacts of warming and fishing*.
Potential to offset increased risks associated with warming by stopping krill fishing (Q3)	“RCP26_20” “RCP26_45” “RCP26_70”	RCP 2.6	Yes, stopped at year denoted in name of scenario	*Assessment of mitigation via cessation of fishing under RCP 2*.*6*.
	“RCP85_20” “RCP85_45” “RCP85_70”	RCP 8.5	Yes, stopped at year denoted in name of scenario	*Assessment of mitigation via cessation of fishing under RCP 8*.*5*.

We developed several scenarios to assess the marginal impacts associated with warming and fishing (summarized in Table 1). First, GGP estimates from RCP 2.6 and RCP 8.5 in an unfished system (“RCP26” and “RCP85”) were used to assess the marginal ecosystem impacts of warming on krill growth alone. To explore how these impacts might interact with those of fishing, we implemented three other scenarios: one with fishing alone (“Fish”), and two others including both fishing and warming (“RCP26_Fish” and “RCP85_Fish”). Finally, we evaluated whether the increased ecosystem risks associated with warming can be offset by ending krill fishing activity in the model after 20 years (“RCP26_20” and “RCP85_20”), 45 years (“RCP26_45” and “RCP85_45”), and 70 years (“RCP26_70” and “RCP85_70”).

In addition to computing marginal impacts, we followed the convention used by previous work [13, 25] and computed marginal “depletion risks.” Within each scenario, we calculated the percentage of simulations in which krill biomass and predator abundances, averaged over the final 30 years, fell below a “depletion threshold.” This threshold was defined as 75% of the respective final biomass (krill) or abundances (predators) in reference simulations with the same model parameterization.

Results are therefore presented as these two metrics, marginal impacts and depletion risks, for each species group. Both are provided first as overall trends across all SSMUs (SSMUs were equally weighted within each species group), and then by SSMU. The two spatial scales are reported to assess whether overall trends were representative of those at smaller scales.

## Results

### Q1. How might the effects of rising temperature on krill growth affect krill biomass and the abundance of krill predators in the Scotia Sea?

**Fig 2:**
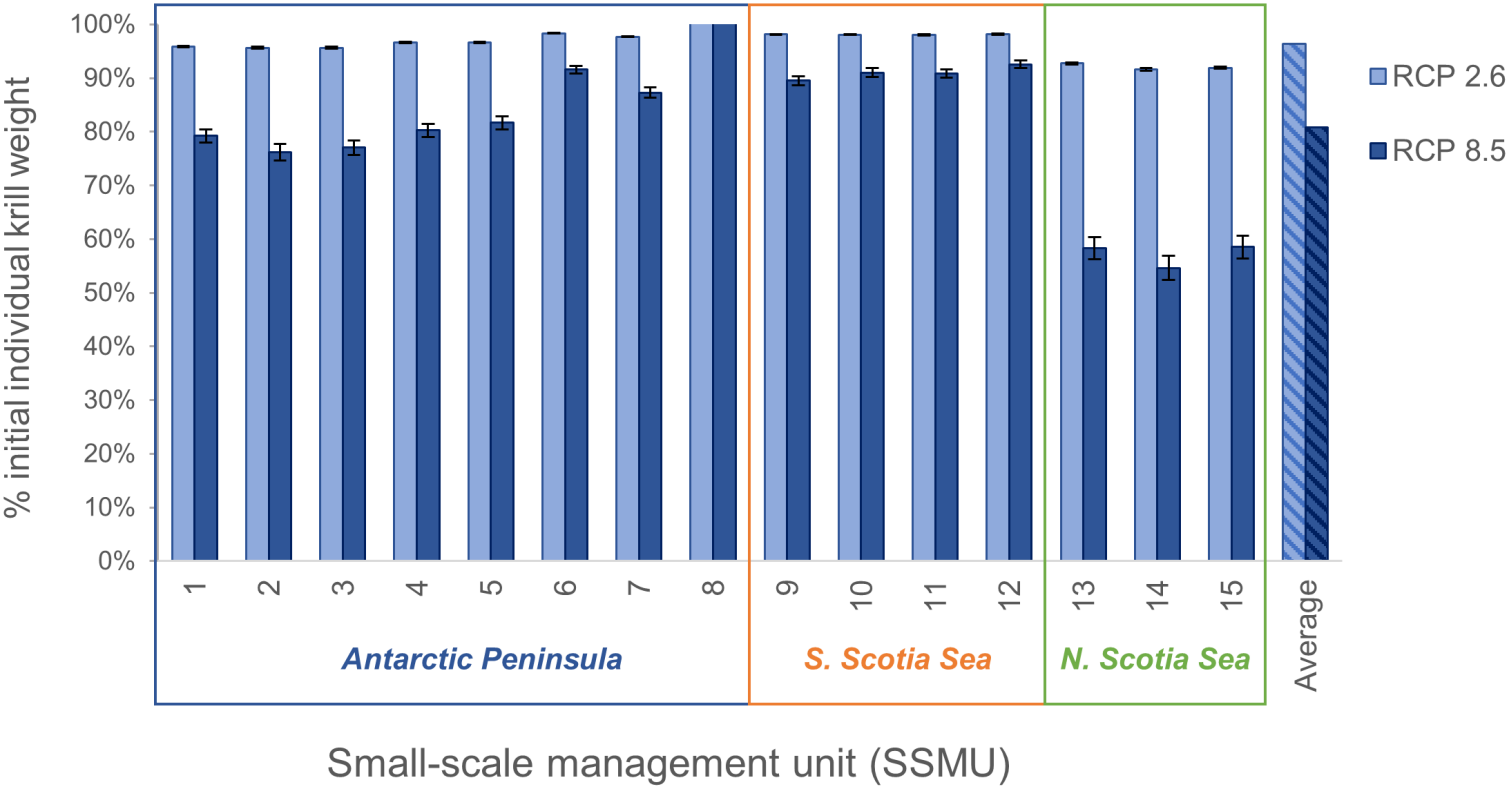
Effects of rising temperature on krill biomass by SSMU. Average change (over the 21^st^ century) in mass of individual krill (*wbar*) under RCP 2.6 (lighter blue) and 8.5 (darker blue) as a percentage of the original mass (0.46g) by SSMU. Current subarea catch limits apply to each of the three boxes indicating the Antarctic Peninsula (SSMUs 1–8), and the southern (SSMUs 9–12) and northern (SSMUs 13–15) Scotia Sea, which are included to highlight large-scale differences in growth potential. The change in individual mass averaged across all SSMUs is at far right (hatched colors). Error bars represent the standard deviation from the mean across simulations in a scenario, capturing the uncertainty introduced by the four parameterizations.

**Fig 3:**
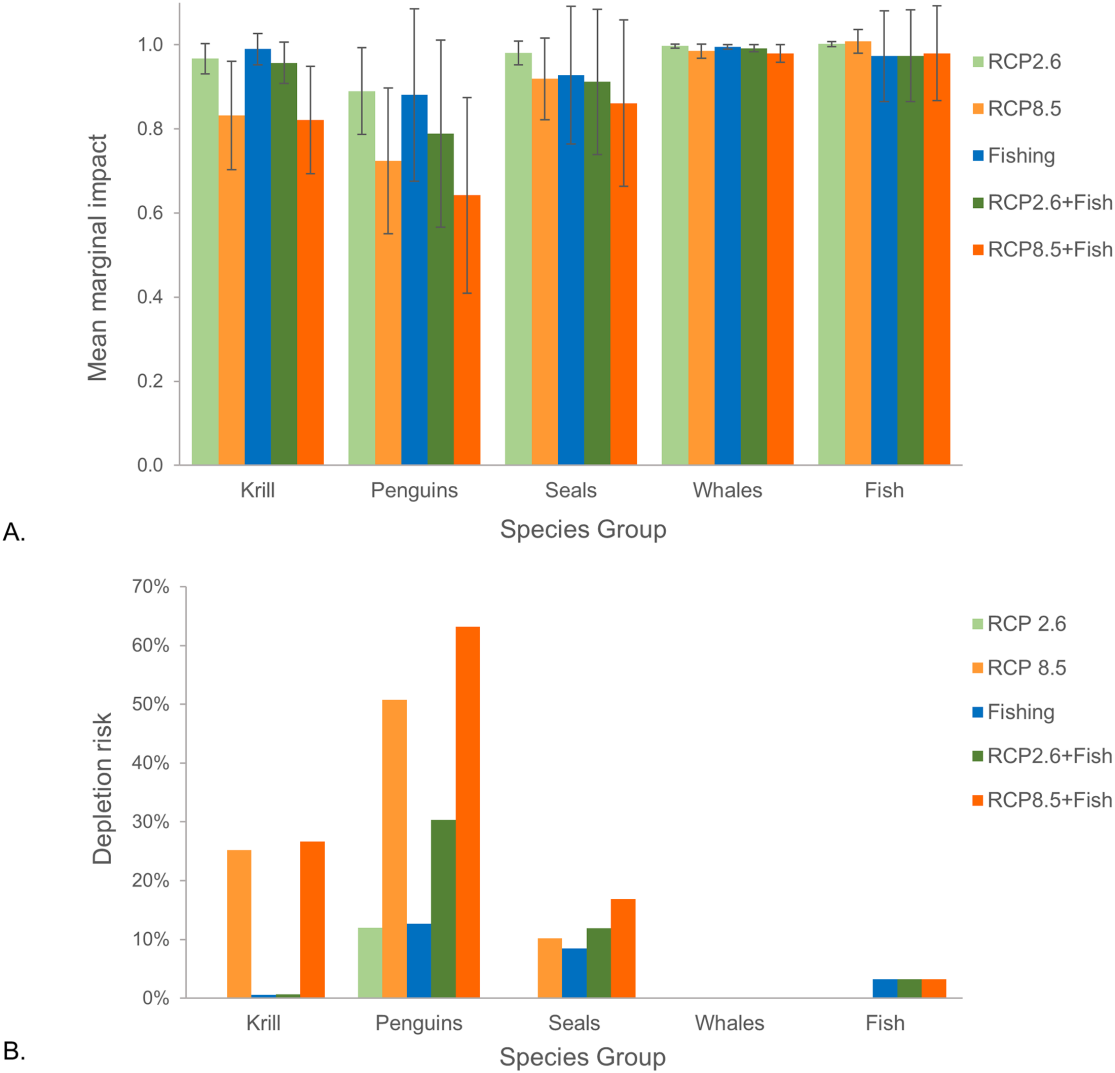
Overall outcomes of warming for krill and their predators. Marginal impacts and risks for krill and krill-dependent predators given the effects of warming on krill growth (RCP 2.6 in green shades, RCP 8.5 in orange shades), fishing (blue), and both warming and fishing (darker green and darker orange), as modeled over the 21^st^ century. The top panel shows mean marginal impact (i.e., the ratio of average krill biomass or predator abundance over the last 30 years of each simulation, 2069–2099, relative to an analogous simulation from the reference scenario), and the bottom panel, B, depletion risk for krill and their predators (i.e. the risk falling below 75% of biomass or abundance in the reference scenario, again averaged over final 30 years). Results are the mean proportion (A) and total percentage (B) across all SSMUs. Error bars in A represent the standard deviation from the mean across all simulations in a scenario, capturing the uncertainty introduced by the four parameterizations.

Modelled effects of warming on krill growth reduced the mass of individual krill relative to the reference scenario. Across the Scotia Sea, the projected mass of individual krill declined on average almost 5% under RCP 2.6, and 19% under RCP 8.5, with notable variation among SSMUs (Fig 2). For both pathways, declines were greatest in the northern Scotia Sea (SSMUs 13–15), least in the southern Scotia Sea (SSMUs 9–12), and differed by SSMU around the Antarctic Peninsula (SSMUs 1–8). For RCP 8.5, the decline was ≥40% throughout the northern Scotia Sea, about 10% in the southern Scotia Sea, and between 0% and about 25% around the Antarctic Peninsula. SSMU-specific declines were smaller for RCP 2.6. These changes in the average mass of individual krill translated to reductions in total krill biomass under both RCPs (Fig 3A), and, under RCP 8.5, a 25% chance that krill biomass would fall below the 75% depletion threshold (Fig 3B).

The projected reductions in average individual krill mass and krill population biomass affected krill-dependent predator populations in our model. These effects were relatively minor under RCP 2.6, but larger under RCP 8.5 (Fig 3). They also varied across predators, with penguins the most impacted. Under RCP 8.5, the abundances of penguins averaged roughly 70% of the reference scenario (Fig 3A), and penguin populations fell below the 75% depletion threshold about 50% of the time (Fig 3B). Climate-driven changes in krill biomass had smaller effects on seals. Under RCP 8.5, abundances of seals averaged roughly 90% of the reference scenario (Fig 3A), and seal populations fell below the 75% threshold about 10% of the time (Fig 3B). Impacts to whale and fish populations were minimal under either climate-change pathway (Fig 3).

**Fig 4:**
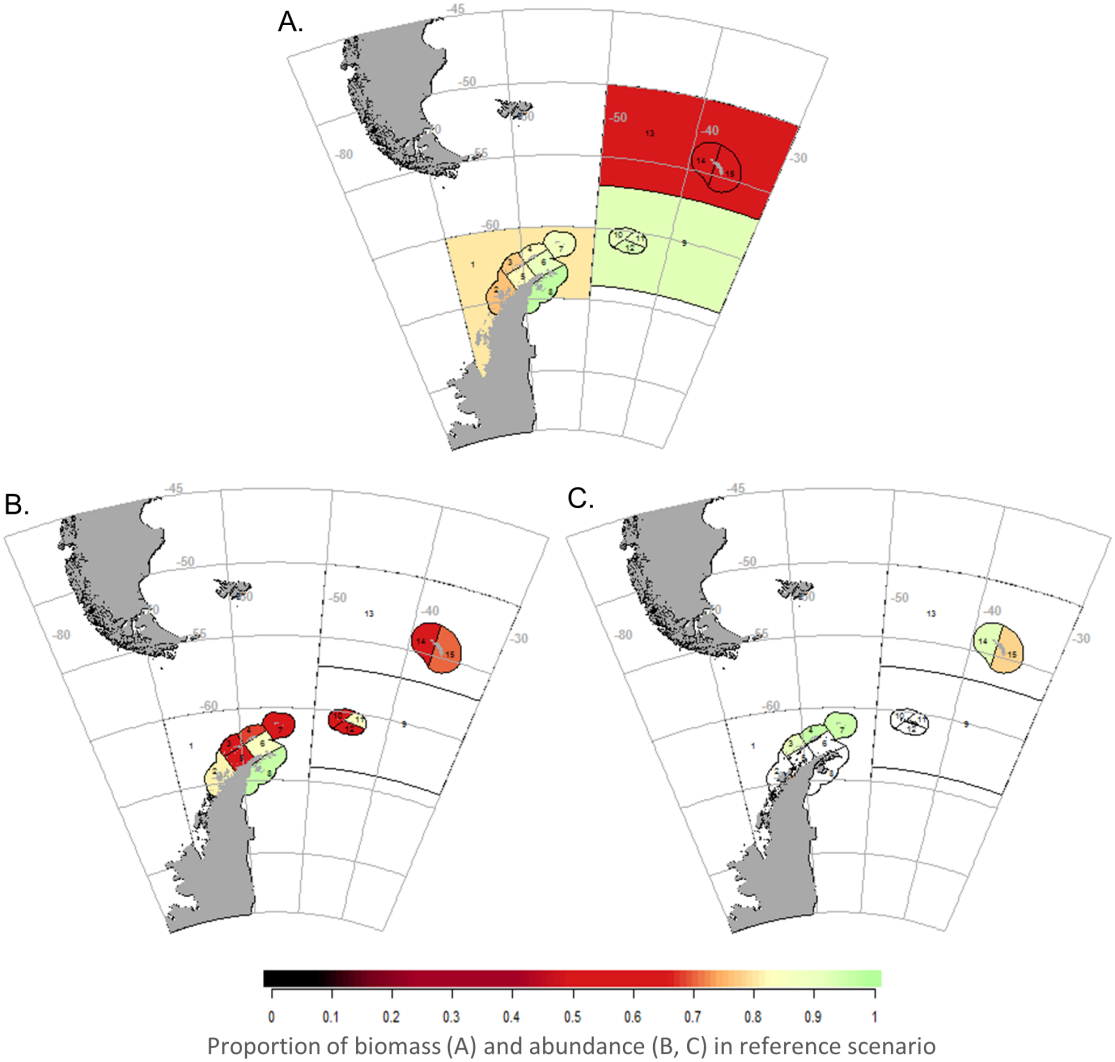
Outcomes of warming for krill, penguins, and seals by SSMU. Marginal impacts on krill biomass (A), penguin abundance (B), and seal abundance (C), owing to the effects of rising temperature on krill growth from RCP 8.5 by the end of the 21^st^ century. Areas without color indicate species groups are not modeled as recruiting there. Results for RCP 2.6 and for whales and fish under RCP 8.5 are shown in S1 and S2 Figs, respectively.

The model outcomes for krill and some predator populations also exhibited spatial variation, which was more pronounced under RCP 8.5 (Fig 4). SSMU-specific declines in krill biomass followed those in the average mass of individual krill. These were largest in the northern Scotia Sea and differed by SSMU around the Antarctic Peninsula (Fig 4A). Interestingly, those SSMUs with the largest penguin population declines did not map directly to those SSMUs where krill biomass dropped the most (Fig 4B). While penguin populations did decline in the northern Scotia Sea, where impacts on krill were strongest, areas with relatively minor reductions in krill biomass also saw some of the greatest declines in penguin populations (e.g. by 50% to 75% in two SSMUs within the southern Scotia Sea). The most affected seal population was in the northern Scotia Sea (SSMU 15) and declined by roughly 20% (Fig 4C).

### Q2. How do the current, permitted level and spatial distribution of fishing effort affect the ecosystem impacts of rising temperature?

Projecting constant fishing at currently permitted levels through the 21^st^ century resulted in effects that were species-group and SSMU-specific. In the absence of climate-change effects on krill growth, fishing at current levels did little to reduce average krill biomass reference scenario (Fig 3A), and consequently, there were almost no risks that krill biomass would fall below the 75% depletion threshold (Fig 3B). In the same fishing-only scenario, average penguin and seal abundances in some SSMUs exhibited more of a decline than those projected under RCP 2.6 alone, but in all SSMUs the declines in the fishing-only scenario were less than those for RCP 8.5 alone (Fig 3A). With only fishing, there was a c. 10% risk of falling below the 75% depletion threshold for both penguins and seals (Fig 3B). Fishing effects on whales and fish were even smaller.

**Fig 5:**
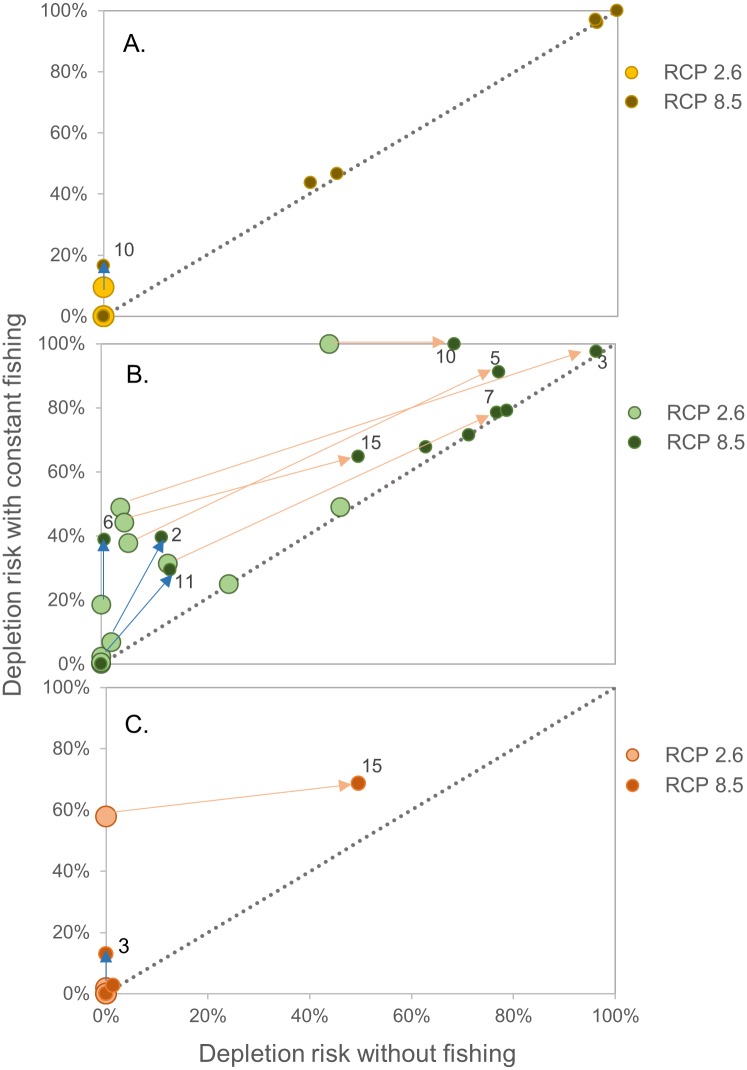
Changes in depletion risk due to fishing for krill, penguins, and seals. Changes in modeled SSMU-specific risk of falling below the 75% depletion threshold (i.e. depletion risk) over the 21^st^ century for krill biomass (A, yellow), penguin abundance (B, green), and seal abundance (C, orange). Larger, light-colored circles represent scenarios for RCP 2.6, and smaller, dark circles are those for RCP 8.5 (note that almost all points for krill under RCP 2.6, larger lighter yellow in A, overlap one another at the origin, indicating no impact). Positive movement along the x-axis between a light circle and a dark circle denotes increased risk from RCP 2.6 to 8.5. Points along the 1:1 line imply similar risk with or without fishing, and positive movement along the y-axis for a light to a dark circle denotes increased risk from fishing. Arrows highlight large changes, and are labeled at the RCP 8.5 point for several SSMUs. Blue arrows indicate increased risk attributed to fishing, and orange arrows indicate increased risk attributed to differences in the climate-change pathways. Equivalent figures for whales and fish are in S3 Fig.

In scenarios with both fishing and warming, the risks of krill biomass dropping below the 75% depletion threshold were mostly not affected by fishing (Fig 3B). The one exception for krill was SSMU 10, which showed larger increases in risk attributed to the presence of fishing than to those attributed to differences between RCP 2.6 and RCP 8.5 (Fig 5A). For penguins, depletion risks increased more due to the presence of fishing than to increased warming in several SSMUs (i.e., 6, 2, and 11), while depletion risks in other SSMUs (i.e., 3, 5, 7, 10, and 15) were greater in response to increased warming than to fishing (Fig 5B). Change in depletion risk for one population of seals (SSMU 15) was greater in response to increased warming, while another saw greater depletion risk with fishing (SSMU 3) (Fig 5C). Collectively, these results suggest that fishing could increase depletion risks in some SSMUs, while the effects of increased warming under RCP 8.5 could be greater than those of fishing in others.

Finally, our results indicated a complex pattern of risk accumulation in the most affected species group, penguins (Table 2). While the average realized declines in penguin abundance under combined effects scenarios were as expected given the individual effects of warming and fishing alone (“Expected” marginal impacts equal to those under “Scenario outcome”), that was not the case for depletion risk. In some areas, scenarios with both fishing and warming projected depletion risks for penguins that were greater than those expected given outcomes for either effect alone (“Expected” versus “Scenario outcome”). That is, the combined effect of warming and fishing on population size (a continuous variable) was linear, but sometimes translated into a nonlinear effect on depletion risk (the average of a binary variable).

**Table 2:** Departure from expected impacts and risks for penguins by small-scale management unit.

	Mean marginal impact						Depletion risk					
SSMU	Scenario outcome (“RCP26_Fish”)	Expected	*Departure from linear*	Scenario outcome (“RCP85_Fish”)	Expected	*Departure from linear*	Scenario outcome (“RCP26_Fish”)	Expected	*Departure from linear*	Scenario outcome (“RCP85_Fish”)	Expected	*Departure from linear*
**1**												
**2**	0.90	0.89	0.01	0.78	0.78	0.00	0.02	0.00	0.02	**0.40**	**0.12**	**0.28**
**3**	0.75	0.73	0.02	0.50	0.51	-0.01	**0.49**	**0.19**	**0.30**	0.98	0.97	0.01
**4**	0.90	0.90	0.00	0.68	0.69	-0.01	0.07	0.02	0.05	0.68	0.63	0.05
**5**	0.75	0.74	0.01	0.54	0.55	-0.01	0.38	0.30	0.08	0.91	0.83	0.08
**6**	0.87	0.87	0.00	0.80	0.80	0.00	**0.19**	**0.04**	**0.15**	**0.39**	**0.05**	**0.34**
**7**	0.83	0.83	0.00	0.61	0.61	0.00	**0.31**	**0.13**	**0.18**	0.79	0.77	0.02
**8**	0.97	0.97	0.00	0.96	0.96	0.00	0.00	0.00	0.00	0.00	0.00	0.00
**9**												
**10**	**0.23**	**0.10**	**0.13**	0.19	0.21	-0.02	1.00	0.97	0.03	1.00	0.98	0.02
**11**	0.88	0.88	0.00	0.82	0.82	0.00	0.00	0.00	0.00	**0.29**	**0.13**	**0.16**
**12**	0.76	0.74	0.02	0.57	0.55	0.02	0.49	0.46	0.03	0.79	0.79	0.00
**13**												
**14**	0.86	0.86	0.00	0.66	0.65	0.01	0.25	0.25	0.00	0.72	0.72	0.00
**15**	0.76	0.76	0.00	0.59	0.60	-0.01	**0.44**	**0.15**	**0.30**	**0.65**	**0.55**	**0.10**

Results for penguins by SSMU in terms of mean marginal impact (left) and depletion risk (right) relative to the reference case. The “Expected” column denotes the expected outcome if impacts of fishing and warming are linearly additive. For mean marginal impact, expected values are calculated as a combination of the scenarios with fishing or warming alone as 1-((1-“Fish”)+ (1-“RCP”)). For depletion risk, they are 1-(1-“Fish”)*(1-“RCP”). Those SSMUs where the difference between the combined effect and the linear combination of individual effects was greater than 0.10 are in bold.

### Q3. Can increased ecosystem risks of rising temperature be offset by stopping krill fishing?

**Fig 6:**
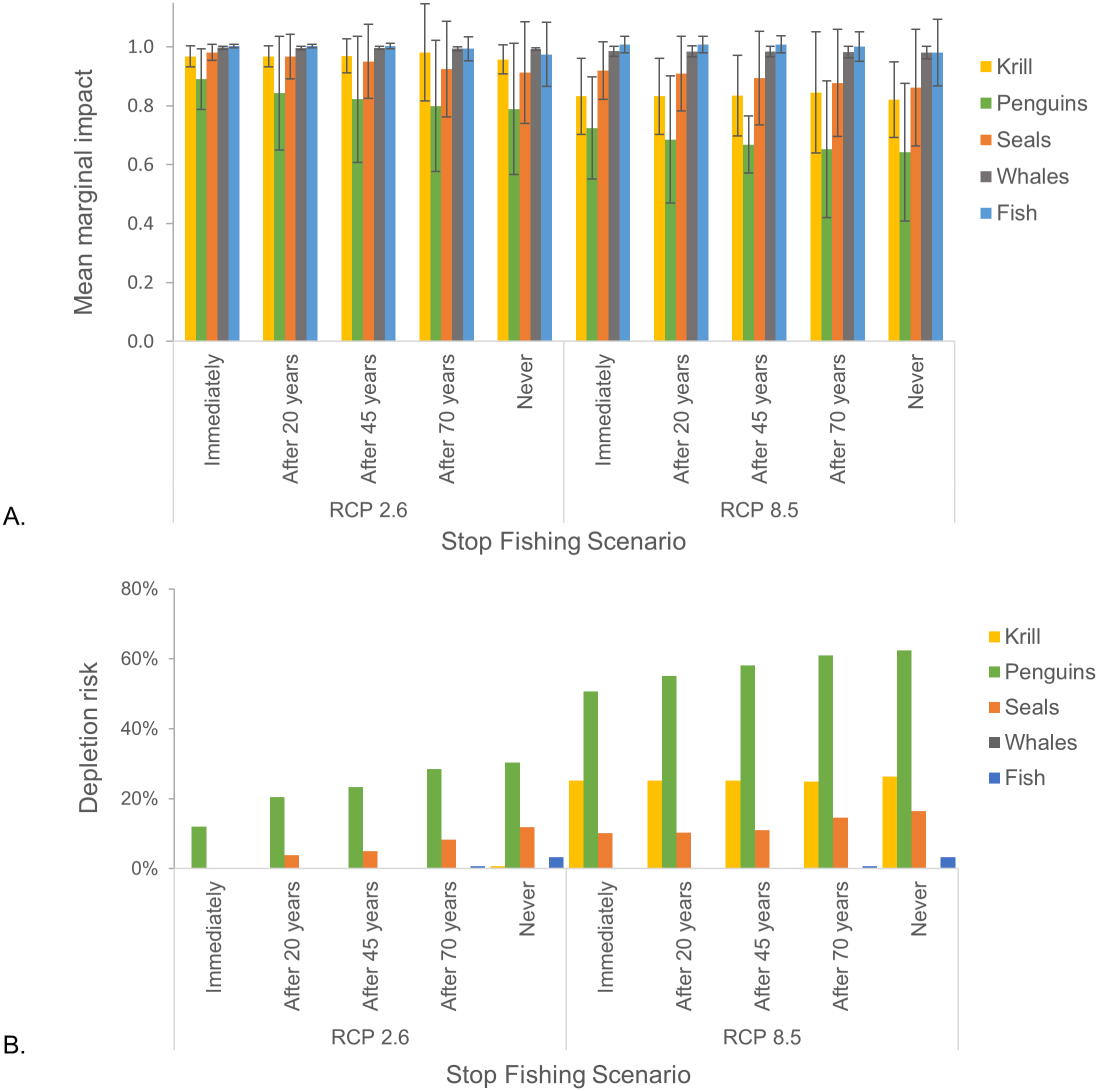
Outcomes of curtailing fishing. Effects of curtailing fishing at various times (i.e. “immediately” and therefore no fishing occurred, cessation at 20, 45, and 70 years into the simulation period, and “never”, or no cessation of fishing) over the 21^st^ century on (A) marginal impacts (i.e. the mean proportion of biomass (krill) or abundance (predators) relative to their respective reference scenario); and (B) the risk of each population falling below the 75% depletion threshold (depletion risk). Results from RCP 2.6 are on the left, and those from RCP 8.5 are on the right for each panel. Error bars in A represent the standard deviation from the mean across all simulations in a scenario, and capture the uncertainty introduced by the four parameterizations.

Curtailing fishing at various points during the simulation period reduced declines in predator abundance, and these reductions were commensurate with the duration of the no-fishing period under both climate-change pathways (Fig 6A). Similarly, depletion risks for predator abundances were reduced when fishing was curtailed sooner (Fig 6B). As fishing had little impact on krill biomass, curtailing fishing was also of little consequence for krill.

**Fig 7:**
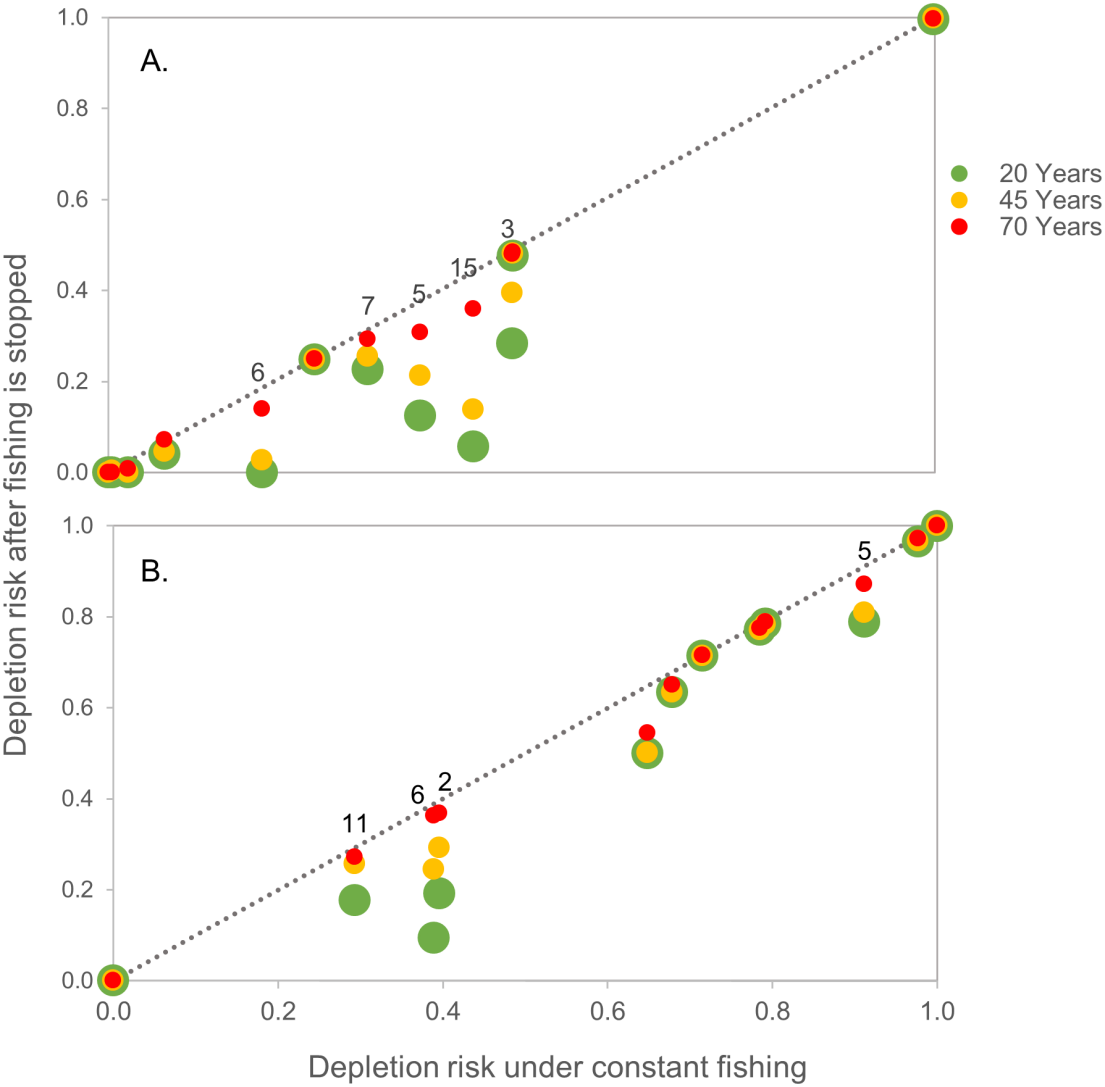
Outcomes of curtailing fishing for penguins by SSMU. The effects of curtailing fishing 20 (green circles), 45 (yellow circles), and 70 (red circles) years into the simulation period on depletion risks given penguins for (A) RCP 2.6 and (B) RCP 8.5. Points along the dashed 1:1 line indicate that curtailing fishing had no effect. Points below the dashed 1:1 line indicate a decrease in depletion risk owing to a cessation in fishing. Populations with discernable differences in risk when fishing was curtailed at different times are labeled at the 70-year point by SSMU.

For some predator groups, the benefits of curtailing fishing varied in space, particularly for penguins (Fig 7). Extended periods without fishing tended to reduce depletion risk under RCP 2.6 for several penguin populations (SSMUs 3, 5, 6, 7, and 15, Fig 7A). However, under RCP 8.5 (Fig 7B), some of these populations (SSMUs 3, 7, and 15) did not benefit from the cessation of fishing, while others (SSMUs 2 and 11) did. Only two penguin populations (SSMUs 5 and 6) benefited from reduced fishing effort under both climate-change pathways. These results were consistent with those described previously in which the risks associated with RCP 8.5 were greater than the risks associated with fishing for some populations, whereas fishing exacerbated the risks posed by RCP 8.5 for other populations (Fig 5).

## Discussion

Our use of a minimally realistic ecosystem model [13] to assess the marginal impacts of one potential consequence of climate change and fishing on the krill-centric ecosystem in the Scotia Sea has several main outcomes. First, the effects of warming on krill growth have implications for krill biomass and the abundances of dependent predators. Second, evaluating aggregated responses across the ecosystem is likely to downplay smaller-scale impacts on some predators, especially penguins. Third, differences in predator responses across SSMUs suggest that some predators and locations may be more vulnerable to changes in the future, and may need specific consideration in management strategies aimed at balancing ecosystem services. Finally, the results indicate that fishing as we implemented it may compound the impacts of warming, and that stopping fishing could help to offset the modeled risks associated with warming for predators in some, but not all, areas.

Growth in marine species is often temperature-dependent and, therefore, one of the major mechanisms by which ocean warming driven by climate change could impact biomass [26]. Our results suggest that warming impacts on krill growth are likely to have negative effects for both the biomass of krill and the abundances of their predators. Current krill catches remain below 50% of the conservative catch limit, yet CCAMLR needs to anticipate a future in which climate change affects the ecosystem and the services it provides. For example, our results demonstrate that the risk of CCAMLR failing to meet a key technical objective for krill (maintaining biomass above 75% of its unimpacted level [14, 27], represented here as the expected biomass in the absence of both climate change and fishing) could increase to c. 25% under RCP 8.5, even in the absence of fishing. Consequently, the risks of seal and penguin populations being depleted below 75% of their unimpacted levels could also increase to c. 10% and c. 51%, respectively. Fishing at 100% of the catch limit, as modeled here, would likely exacerbate those risks.

These aggregated results de-emphasize important outcomes at smaller spatial scales, in this case SSMUs, where fishing, warming, or both might have greater impacts. This finding echoes other work (e.g. [14, 27]), and is an important motivation for using spatially-explicit models like the one here. However, management of marine ecosystems is often focused on larger spatial scales, as it is in the Southern Ocean, because aggregation can simplify both the translation of science to management advice and the implementation of resulting management action. It is therefore important to check whether trends at smaller spatial scales are captured at larger spatial scales. Our results suggest that smaller scale responses to climate change impacts, like ocean warming, can be lost with aggregation. For example, we find spatial separation at the SSMU scale between the strongest effects on krill and the strongest impacts on penguins, even in the absence of fishing. These outcomes arise from the spatial processes represented in the model, including movement (or lack thereof) of krill, and seasonal differences in the foraging distributions of predators. This is a useful demonstration that local food-web structure is affected by processes operating at multiple scales, including global climate change and changes elsewhere in the same ecosystem [28].

We also found differences between species groups in our simulations, with whales and fish largely unaffected by warming effects on krill growth, and penguins the most sensitive. Even RCP 2.6, which includes a return to radiative forcing similar to that for 2020 by the end of the 21st century, increased the estimated risks for penguins by c. 12%. Previous analysis using the model here indicates that penguin population responses tend to magnify modelled perturbations [20] due to depensatory dynamics [13]. This formalizes a clear hypothesis that depensatory dynamics might make penguins particularly vulnerable to changes in krill biomass. Identifying whether this is an accurate representation of penguin population dynamics should be a research priority. Other work supplies some evidence that certain species and populations will be more sensitive than others to the effects of ocean warming and changes in krill biomass (e.g. [29]), with penguins particularly susceptible (e.g. [30]).

More broadly, our results have implications for similar ecosystems where dynamics are strongly impacted by a single trophic level, such as that occupied by krill in the Southern Ocean. Previous research demonstrates krill predators tied to land-based breeding colonies may be more vulnerable if changes in available prey occur during the breeding season, as this may cause declines in breeding success [31, 32]. Outcomes may also be exacerbated for krill-dependent predators who forage in areas temporally or spatially disparate from breeding areas (e.g. some cetaceans and seals [33, 34]), and those that require continual and immediate feeding while rearing young (e.g. marine mammals and seabirds, [35]). These examples from the Antarctic demonstrate the potential for ecosystem consequences of changes in one critical prey species, with implications for other similar, or potentially “wasp-waist” [36], ecosystems.

Comprehensive evaluation of all possible climate change impacts on an ecosystem is currently impractical. We have reduced this complexity by focusing on the marginal impacts of a single process, the potential for warming to reduce krill growth. In reality, krill are affected by a range of physical drivers that may be altered by climate change, such as sea-ice extent and temperature [37, 38], as well as increased UV exposure and ocean acidity [29]. Our work does not address these additional drivers; we assumed they would remain unchanged. This will likely not be the case, and alterations in these drivers may have additional effects. For example, changes in primary production might be important for future krill production as well [10, 12]. Climate change will also likely influence krill through processes beyond growth such as recruitment and natural mortality (e.g. [9]), and outcomes may differ across life-history stages [29].

In addition, climate change may have direct impacts on the krill-dependent predators themselves, potentially via a variety of physical drivers, such as changes in sea ice (e.g. [30]), snowfall [39], and warmer conditions (e.g. [40]), with consequences for life history and behavior (e.g., [32, 41, 42]). Outcomes are likely to vary by species and location, will depend on the ability of a species to adapt, and may be confounded by current or previous human harvesting [29]. Overall, climate-change outcomes will be more complicated than those we project here, and are likely to be generally negative for krill-dependent ecosystems [9] (but see [12]).

In terms of fishing, simulated here at the current catch limit and the recent spatial distribution of effort, overall estimated risks were low, on par with those for RCP 2.6. The main exception was for seals, which had zero depletion risk attributable to RCP 2.6 but c. 8% attributable to fishing. The longer fishing period and more recent effort distribution we modelled resulted in higher average risks to penguins and seals than those estimated by Watters et al. [13]. In addition, for penguins, we found nonlinear interactions between fishing and the effects of warming when both were implemented in our model, with results indicating a greater increase in depletion risk than in the overall depletion of abundance (Table 2).

As the outcomes of climate change and fishing are important drivers of change in marine ecosystems, there is great potential for the former to compound the consequences of the latter. Reducing fishing is an important means by which management can reduce this potential in fully- or over-exploited marine ecosystems [5]. We explored this issue by investigating whether total cessation of fishing in an underexploited fishery [14] could offset some of the risks to predators resulting from ocean warming. Total cessation of fishing is a blunt tool to partially mitigate the effects of climate change, with significant costs in terms of lost provisioning services (i.e. fisheries revenue, employment opportunities, and food security). Our results at the system-wide scale suggest fishing cessation would not reduce the risk of CCAMLR failing to meet its technical objective for krill following ocean warming under severe climate change (RCP 8.5). However, simulated stoppages reduced compounded risks to some krill predators, with earlier stoppages resulting in greater risk reductions. The extent of the risk reduction varied with species group and location at smaller spatial scales, where stoppage reduced risk by 20% to 30% for some penguin populations, but made little difference to others.

Whether or not curtailing fishing is an effective mitigation measure depends on the time an ecosystem takes to recover from the effects of fishing. Previous simulations with this model suggest that different species groups recover at different rates following the cessation of krill fishing [27]. Our projections to the end of the current century also suggest such outcomes (S4 Fig). Fish quickly returned to unimpacted levels following a cessation of fishing, but end points for penguins varied depending on when fishing stopped. It is unclear how long it might take all predator groups to recover fully, but our results suggest the potential for lasting effects that compound outcomes of warming long after fishing has ended. This is an area for future research.

The current krill catch limit and spatial distribution of fishing effort imply risk to some krill predators even in the absence of warming, but many predator populations were unaffected by simulated fishing. This suggests that maintaining the current catch limit and preventing any further spatial concentration of effort would help to balance ecosystem services and avoid exacerbating the ecosystem impacts of warming. Regardless, our results provide several insights for managing fishing in the face of future climate change. First, effective management needs to address a range of spatial scales [43, 44]. Second, there is a need to identify vulnerable predator populations, for example using remote sensing of penguin colonies [41], and devise targeted management measures to protect them. Finally, disparity in both the risks posed by fishing and the potential for a cessation of fishing to reduce risks indicates that spatially structured management may simultaneously mitigate risks for the most vulnerable populations while relaxing constraints on the krill fishery where populations are more robust. Doing so might provide flexibility for balancing trade-offs between ecosystem services that regional-scale policies may not.

CCAMLR’s management approach includes spatial measures and a desire for feedback-management [14, 15], tactics that exemplify best practices in regional fisheries management [45, 46]. Targeted actions might therefore include fine-scale catch restrictions, which could vary in response to ecosystem monitoring data. As predator populations are influenced by processes occurring elsewhere in the Antarctic ecosystem and beyond, further investigation will be necessary to identify the appropriate locations for such targeted measures. These investigations might also identify locations where catch limits can be relaxed without increasing risks to predators. However, our results also imply that the risks for krill and their predators resulting from ocean warming cannot fully be offset by stopping fishing.

We aimed to understand how a warming global ocean under climate change may influence a regional social-ecological system, and conclude that future management of such systems must account for spatial heterogeneity in risk. Our results point to the potential utility of spatial and feedback management to mitigate outcomes for the most vulnerable populations, while maintaining other ecosystem services important for people. Management under shifting conditions in the future will be challenging, but current mandates, governance structures, and research in the Antarctic support novel endeavors to ensure management effectiveness, and CCAMLR has an opportunity to lead the way on effectively balancing ecosystem services in a changing climate.

## Supporting information

S1 Fig Outcomes of ocean warming for krill and their predators under RCP 2.6 by small-scale management unit. Marginal impacts on krill biomass (A) and penguin (B), seal (C), whale (D), and fish (E) abundance owing to the effects of ocean warming on krill growth from RCP 2.6 by the end of the 21^st^ century. Areas without color indicate species groups are not modeled as recruiting there.
(TIF)

S2 Fig Outcomes of ocean warming impacts on krill growth for whales and fish under RCP 8.5 by small-scale management unit. Marginal impacts on whale (A) and fish (B) abundance owing to the effects of ocean warming on krill growth from RCP 8.5 by the end of the 21^st^ century. Areas without color indicate species group are not modeled as recruiting there.
(TIF)

S3 Fig Changes in depletion due to fishing for whales and fish. Changes in modeled SSMU-specific risk of falling below the 75% depletion threshold (i.e. depletion risk) over the 21^st^ century for whale (A, grey) and fish (B, blue) abundance owing to scenarios with and without fishing for the RCP 2.6 (large, light circles) and RCP 8.5 (small, dark circles) scenarios. Positive movement from light to dark circles along the x-axis denotes increased risk from RCP 2.6 to 8.5. Points along the 1:1 line imply similar risk with or without fishing, with positive movement from light to dark circles along the y-axis denoting increased risk from fishing. All points for whales and most for fish overlap at the origin, indicating no impact.
(TIF)

S4 Fig Projected annual marginal impacts to assess lasting impacts of fishing on predator groups. The annual marginal impacts plotted each year (x-axis) across model scenarios when fishing is stopped immediately (green), after 20 years (orange), after 45 years (gray), after 70 years (yellow), or never, for penguins (A), seals (B), whales (C), and fish (D). Left column is for RCP 2.6, and right is RCP 8.5.
(TIF)

S1 Table Proportional distributions of krill catch by SSMU. Proportions used in earlier versions of the model are under “Previous Model” (Table 2 in [13]). The updates used here, “Current Model”, are derived from catches taken during the 2009–2015 fishing seasons and under limits at finer spatial scales specified by current management [16]. Both include the proportional distribution of fishing across SSMUs (“Annual Distribution”), and then how this proportion was distributed seasonally within each SSMU (“Seasonal Distribution”). Therefore, annual distribution sums to one by column, seasonal distribution by row (“Summer” + “Winter”).
(DOCX)
