# Supplementary figures and images for: Impacts of rising sea temperature on krill increase risks for predators in the Scotia Sea
Source: PLoS One. 2018 Jan 31;13(1):e0191011. doi: 10.1371/journal.pone.0191011 (PMC5791976; doi:10.1371/journal.pone.0191011)

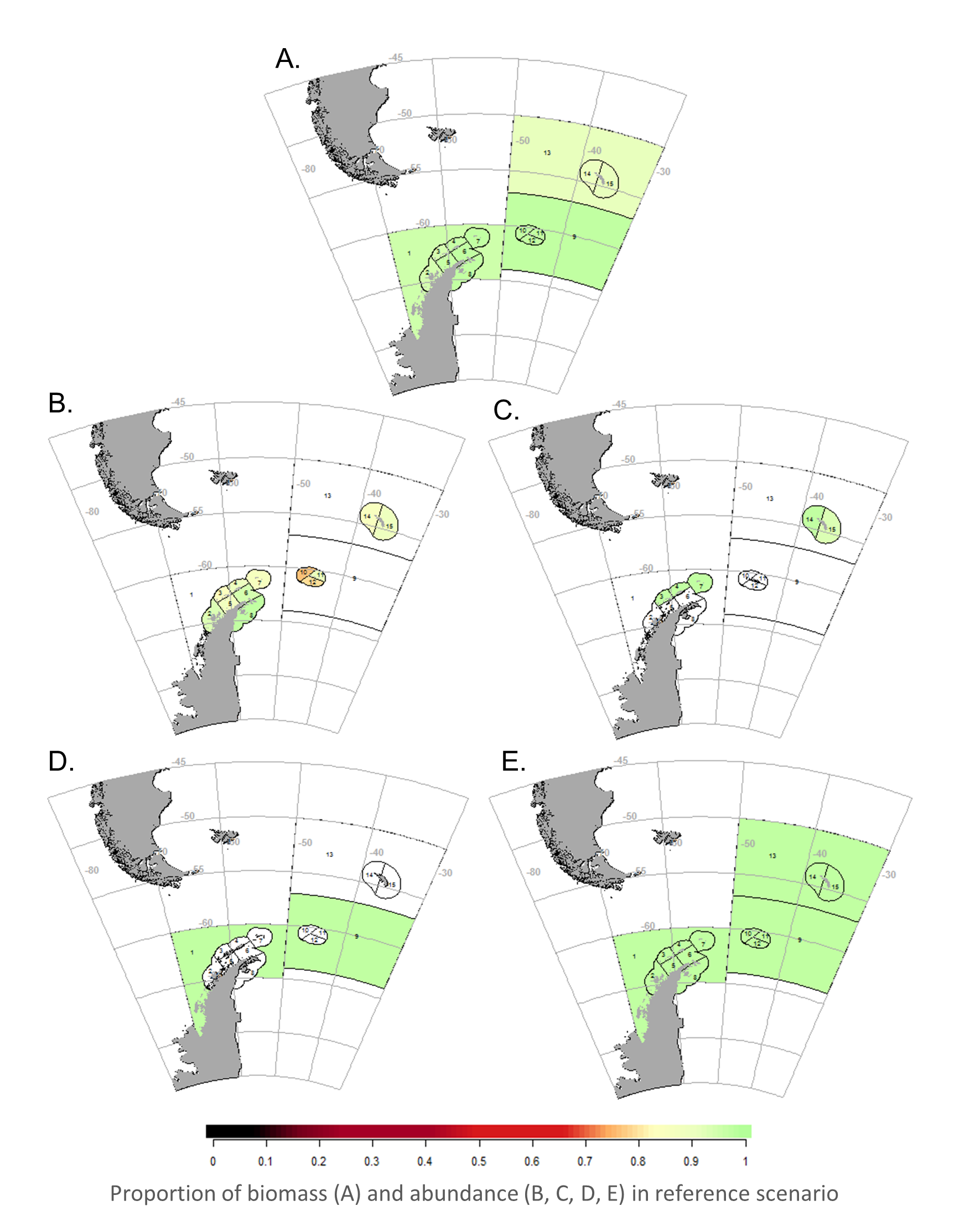

Supplement: S1 Fig — Marginal impacts on krill biomass (A) and penguin (B), seal (C), whale (D), and fish (E) abundance owing to the effects of ocean warming on krill growth from RCP 2.6 by the end of the 21st century. Areas without color indicate species groups are not modeled as recruiting there. (TIF) [file pone.0191011.s001.tif]

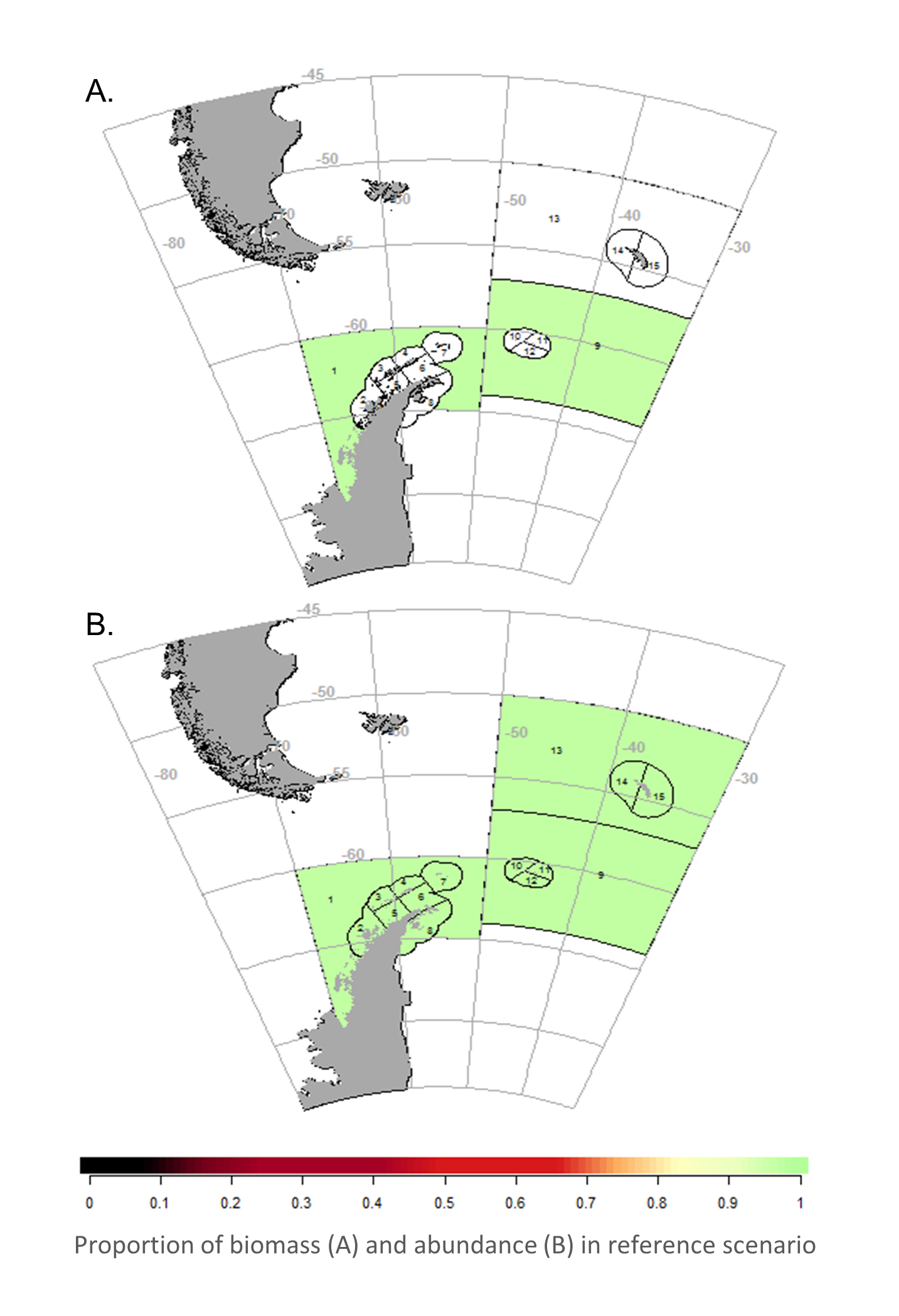

Supplement: S2 Fig — Marginal impacts on whale (A) and fish (B) abundance owing to the effects of ocean warming on krill growth from RCP 8.5 by the end of the 21st century. Areas without color indicate species group are not modeled as recruiting there. (TIF) [file pone.0191011.s002.tif]

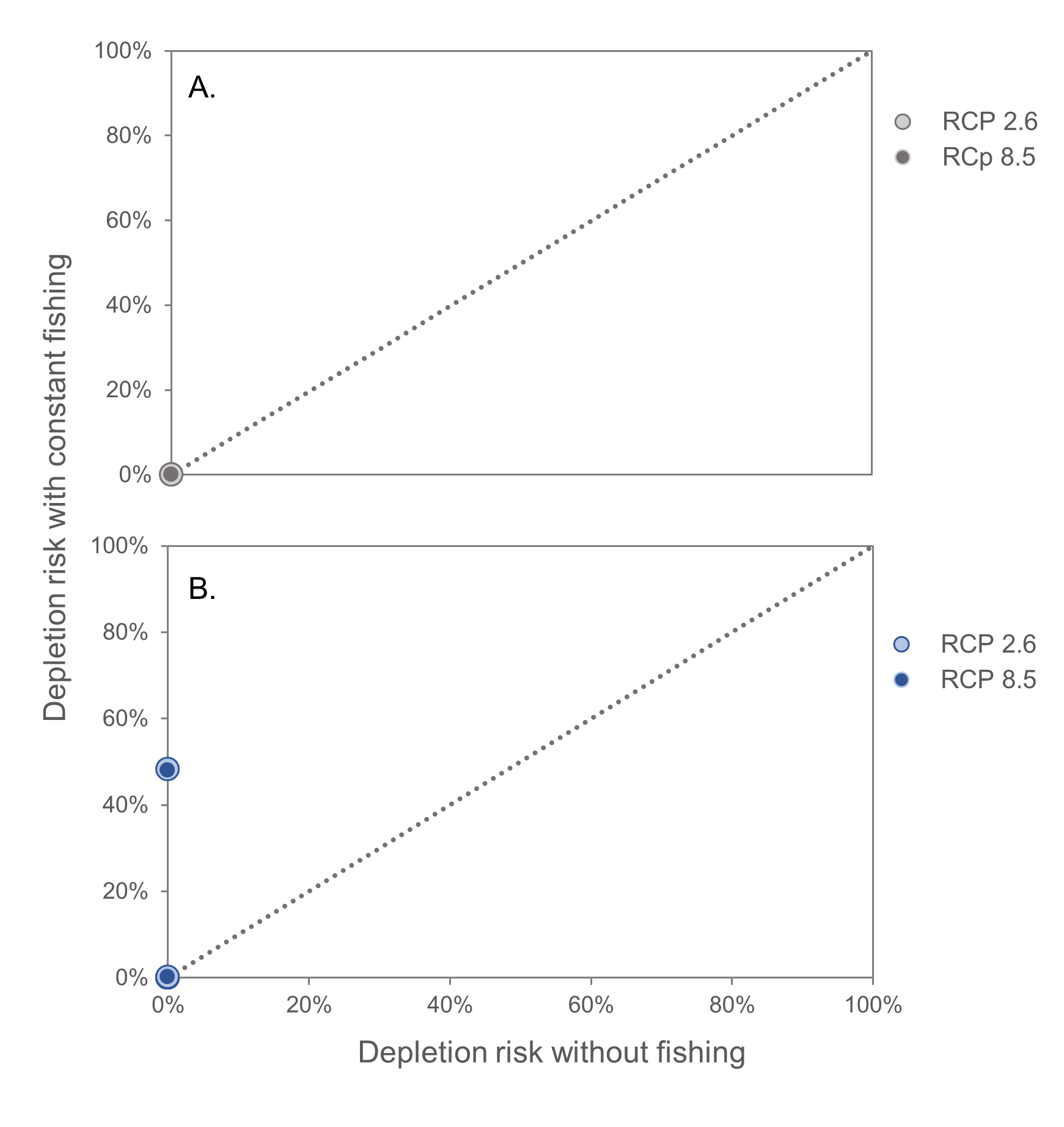

Supplement: S3 Fig — Changes in modeled SSMU-specific risk of falling below the 75% depletion threshold (i.e. depletion risk) over the 21st century for whale (A, grey) and fish (B, blue) abundance owing to scenarios with and without fishing for the RCP 2.6 (large, light circles) and RCP 8.5 (small, dark circles) scenarios. Positive movement from light to dark circles along the x-axis denotes increased risk from RCP 2.6 to 8.5. Points along the 1:1 line imply similar risk with or without fishing, with positive movement from light to dark circles along the y-axis denoting increased risk from fishing. All points for whales and most for fish overlap at the origin, indicating no impact. (TIF) [file pone.0191011.s003.tif]

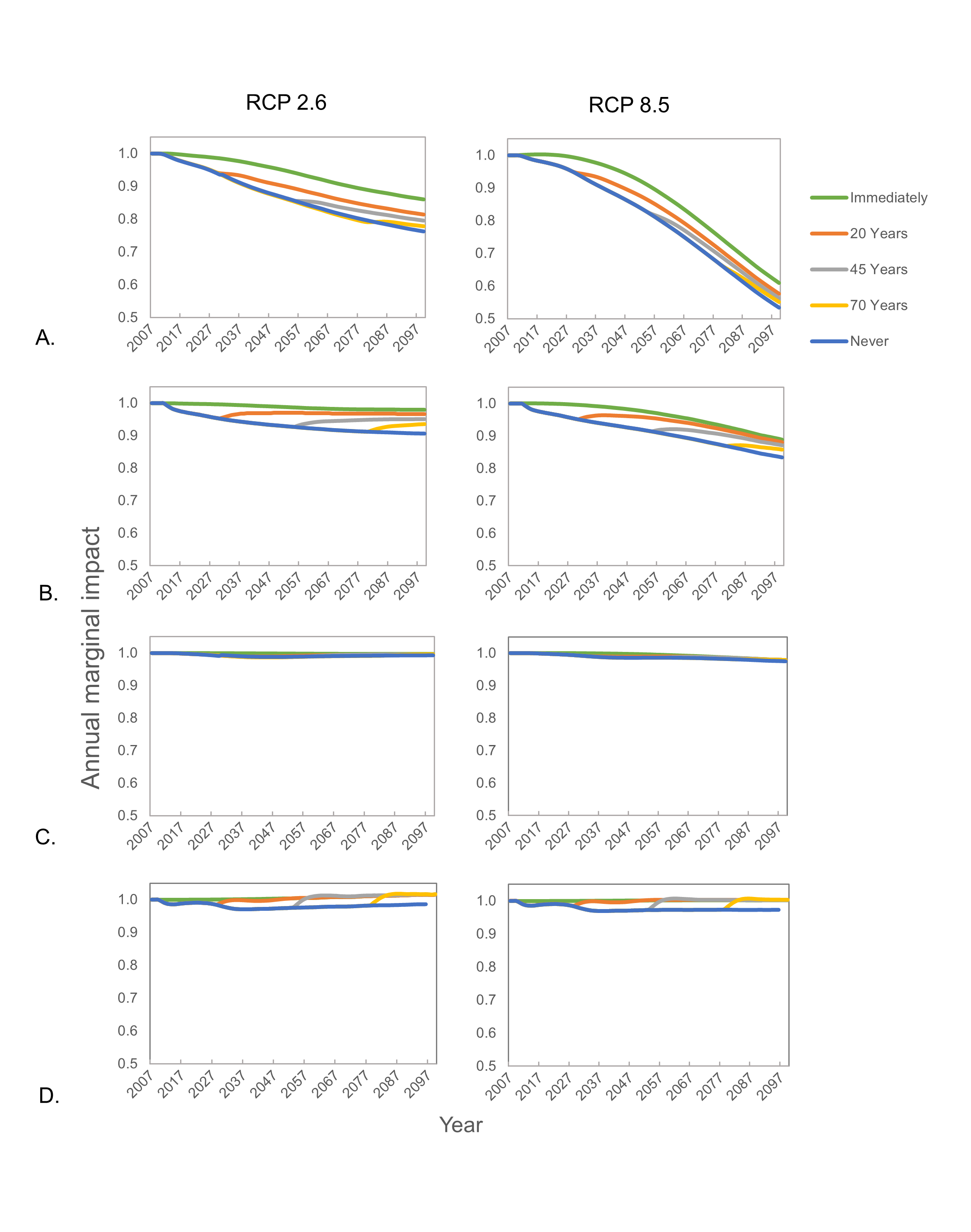

Supplement: S4 Fig — The annual marginal impacts plotted each year (x-axis) across model scenarios when fishing is stopped immediately (green), after 20 years (orange), after 45 years (gray), after 70 years (yellow), or never, for penguins (A), seals (B), whales (C), and fish (D). Left column is for RCP 2.6, and right is RCP 8.5. (TIF) [file pone.0191011.s004.tif]
