# Supplementary material for: Impacts of rising sea temperature on krill increase risks for predators in the Scotia Sea
Source: PLoS One. 2018 Jan 31;13(1):e0191011. doi: 10.1371/journal.pone.0191011 (PMC5791976; doi:10.1371/journal.pone.0191011)
Supplement: S1 Table — Proportions used in earlier versions of the model are under “Previous Model” (Table 2 in [13]). The updates used here, “Current Model”, are derived from catches taken during the 2009–2015 fishing seasons and under limits at finer spatial scales specified by current management [16]. Both include the proportional distribution of fishing across SSMUs (“Annual Distribution”), and then how this proportion was distributed seasonally within each SSMU (“Seasonal Distribution”). Therefore, annual distribution sums to one by column, seasonal distribution by row (“Summer” + “Winter”). (DOCX) [file pone.0191011.s005.docx]

## “Impacts of rising sea surface temperatures on krill increase risks for predators in the Scotia Sea.”

Emily S. Klein, Simeon L. Hill, Jefferson T. Hinke, Tony Phillips, and George M. Watters.

## Supporting Information

### Updates to fishing effort

To best represent the currently permitted, conservative level of fishing with our ecosystem model, we used the average recent distribution of fishing catch (2009 to 2015). This was employed to simulate catch among SSMUs annually, as well as within each SSMU seasonally (Table SI).

### Additional results

We assessed outcomes of climate change impacts on krill growth for both krill and their dependent predators across two climate change pathways (RCP 2.6 and RCP 8.5, see Methods section) and under fishing for Antarctic krill (*Euphausia superba*). Additional results to those in the main text are provided here. First, RCP 2.6 alone had little impact, which is as expected given that this scenario projects greenhouse gas concentrations reducing by the end of the century, and therefore concurrent recovery in krill growth (S1 Fig). Second, impacts did not cause discernable changes in populations of fish or whales, under either RCP 2.6 (S1 Fig) or RCP 8.5 (S2 Fig). The conservative fishing implemented here also had little impact (see Fig 3 in the main text), and there was little or no interaction between fishing and climate change for most whale or fish populations (S3 Fig). The only population showing an impact is fish in SSMU 10. This outcome is due to fishing, as evidenced by it being away from the 1:1 line, and is unchanged by climate pathway.

To explore whether fishing had long-term effects that may compound those of rising SST on krill growth, we also assessed marginal impacts on an annual basis to determine if populations continued to experience consequences after fishing had been stopped in the model (S4 Fig). While it appears that some species groups do recover prior to the end of the century in the model (e.g. whales and fish, S4 Fig, C and D), others show a lasting impact (seals and penguins, S4 Fig, A and B).

**Table SI**. **Proportional distributions of krill catch for each of the 15 SSMUs.**

| **SSMU** | **Previous model** | | | **Current model** | | |
| --- | --- | --- | --- | --- | --- | --- |
|  | Annual Distribution | Seasonal Distribution | | Annual Distribution | Seasonal Distribution | |
|  |  | *Summer* | *Winter* |  | *Summer* | *Winter* |
| 1 | 0.010 | 1.000 | 0.000 | 0.001 | 0.785 | 0.215 |
| 2 | 0.000 | 1.000 | 0.000 | 0.030 | 0.442 | 0.558 |
| 3 | 0.130 | 1.000 | 0.000 | 0.022 | 0.447 | 0.553 |
| 4 | 0.040 | 1.000 | 0.000 | 0.013 | 0.759 | 0.241 |
| 5 | 0.010 | 1.000 | 0.000 | 0.120 | 0.302 | 0.699 |
| 6 | 0.010 | 1.000 | 0.000 | 0.060 | 0.371 | 0.629 |
| 7 | 0.040 | 1.000 | 0.000 | 0.004 | 0.835 | 0.165 |
| 8 | 0.000 | 1.000 | 0.000 | 0.001 | 0.732 | 0.268 |
| 9 | 0.010 | 1.000 | 0.000 | 0.004 | 0.858 | 0.143 |
| 10 | 0.320 | 1.000 | 0.000 | 0.427 | 0.482 | 0.518 |
| 11 | 0.020 | 1.000 | 0.000 | 0.011 | 0.963 | 0.037 |
| 12 | 0.020 | 1.000 | 0.000 | 0.000 | 0.527 | 0.473 |
| 13 | 0.010 | 0.000 | 1.000 | 0.000 | 0.743 | 0.257 |
| 14 | 0.080 | 0.000 | 1.000 | 0.013 | 0.071 | 0.929 |
| 15 | 0.300 | 0.000 | 1.000 | 0.294 | 0.011 | 0.989 |

Proportions used in earlier versions of the model are under “Previous Model” (Table 2 in [13]). The updates used here, “Current Model”, are derived from catches taken during the 2009-2015 fishing seasons and under limits at finer spatial scales specified by current management [16]. Both include the proportional distribution of fishing across SSMUs (“Annual Distribution”), and then how this proportion was distributed seasonally within each SSMU (“Seasonal Distribution”). Therefore, annual distribution sums to one by column, seasonal distribution by row (“Summer” + “Winter”).
